# A meta-analysis of the association of ApaI, BsmI, FokI, and TaqI polymorphisms in the vitamin D receptor gene with the risk of polycystic ovary syndrome in the Eastern Mediterranean Regional Office population

**DOI:** 10.18502/ijrm.v20i6.11439

**Published:** 2022-07-06

**Authors:** Arvin Shahmoradi, Abbas Aghaei, Kimya Ghaderi, Mohammad Jafar Rezaei, Asaad Azarnezhad

**Affiliations:** ^1^Department of Laboratory Medicine, Faculty of Paramedical, Kurdistan University of Medical Sciences, Sanandaj, Iran.; ^2^Social Determinants of Health Research Center, Research Institute for Health Development, Kurdistan University of Medical Sciences, Sanandaj, Iran.; ^3^Department of Anatomical Sciences, Faculty of Medicine, Kurdistan University of Medical Sciences, Sanandaj, Iran.; ^4^Student Research Committee, Kurdistan University of Medical Sciences, Sanandaj, Iran.

**Keywords:** Meta-analysis, Polycystic ovary syndrome, Polymorphisms, Vitamin D receptor.

## Abstract

**Background:**

The results of case-control studies on the association between vitamin D receptor gene (*VDR*) polymorphisms and polycystic ovary syndrome (PCOS) are inconclusive.

**Objective:**

We aimed to more precisely evaluate the correlation between the ApaI, BsmI, FokI, and TaqI *VDR* gene polymorphisms and PCOS susceptibility.

**Materials and Methods:**

PubMed, Scopus, Science Citation Index, and Google Scholar databases were searched to retrieve related reports released up to the end of 2020. To evaluate the association strength of the *VDR* gene polymorphisms with PCOS risk, pooled odds ratios (OR) with a 95% confidence interval were determined.

**Results:**

In total, 1,119 subjects (560 PCOS cases and 559 controls) from 7 studies were included which met the inclusion criteria. A statistically significant association between the TaqI polymorphism and PCOS susceptibility was found in the Eastern Mediterranean Regional Office population (T vs. t: OR = 0.715; TT vs. tt: OR = 0.435, p < 0.001; TT vs. Tt+tt: OR = 0.696, p = 0.01; tt vs. TT+Tt: OR = 1.791, p < 0.001). It was found that the ApaI variant was a risk factor in the dominant inheritance model (AA vs. Aa+aa: OR = 1.466, p = 0.01) and the FokI polymorphism was a protective factor in the recessive inheritance model (ff vs. FF+Ff: OR = 0.669, p = 0.04). The *VDR* BsmI polymorphism did not show association with PCOS susceptibility.

**Conclusion:**

Our meta-analysis revealed that the *VDR* ApaI in the dominant model, *VDR* FokI in the recessive model, and *VDR* TaqI polymorphisms in all genetic models are associated with vulnerability to PCOS. However, further studies with a larger sample size are required.

## 1. Introduction

Polycystic ovary syndrome (PCOS) is one of the most common syndromes in reproductive-age people, distinguished by clinical characteristics such as prolonged anovulation, menstrual dysfunction, and polycystic ovaries (1, 2). According to the National Institutes of Health 1990 criteria and the Rotterdam 2003 criteria, the combined prevalence of PCOS is ∼ 4-21% worldwide (3). Women with PCOS have a high probability of type 2 diabetes mellitus and cardiovascular disease (4, 5). Patients with PCOS have an impaired glucose tolerance, insulin resistance, hyperinsulinemia, and obesity (6, 7). However, the exact etiology and underlying pathological mechanisms of PCOS are not clear.

Interactions between genetic and environmental factors are believed to play a significant role in the incidence and progression of PCOS (8, 9, 10). Insulin resistance and hyperinsulinemia are recurrent metabolic disturbances in PCOS. Research demonstrates that levels of vitamin D could be related to hormonal and metabolic conditions (11). Vitamin D is converted to 1, 25-dihydroxycholecalciferol in the kidneys and liver (12, 13). Lines of evidence suggest a significant relationship of vitamin D levels with the pathogenesis, signs, and symptoms of PCOS (14). A recent meta-analysis identified more considerable variation in serum 25-hydroxyvitamin D, total cholesterol, serum insulin, low-density lipoprotein cholesterol, and triglycerides in women with PCOS than in healthy subjects (15).

**Figure 1:**
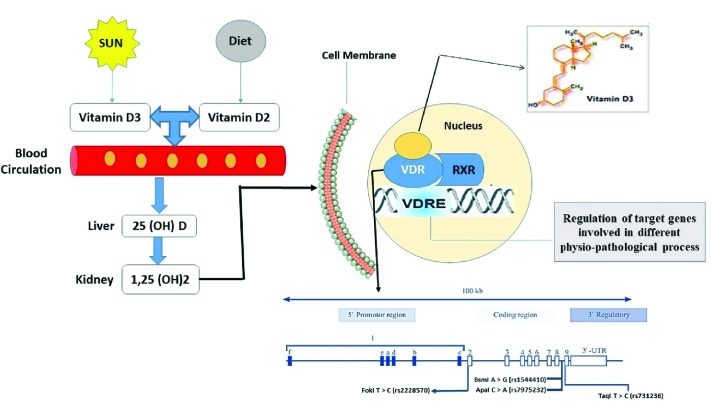
A flow diagram of *VDR* gene function in the cell and the related SNPs that could be involved in the susceptibility to PCOS.

The vitamin D receptor (*VDR*), a ligand-dependent transcription factor belonging to the steroid/thyroid hormone receptor superfamily, is responsible for vitamin D's effect. *VDR* is commonly found in many tissues of the female reproductive system and regulates the biological effects of vitamin D (16, 17). As is illustrated in figure 1, *VDR* and unoccupied retinoid X receptor form a heterodimer transcription unit which regulates the transcription through binding to the vitamin D response element in the promoter region of target genes (18, 19). About 3% of the human genome, including genes essential to glucose metabolism, are regulated by *VDR* (13).

The *VDR* gene is located on chromosome 12q13.11 and contains 14 exons that provide instruction for a 427-amino-acid protein (20). The *VDR* gene contain 4 mostly reported single nucleotide polymorphisms (SNPs) including FokI (rs10735810) in exon 2, ApaI (rs7975232) in intron 8, TaqI (rs731236) in exon 9, and BsmI (rs1544410)in intron 8(21)*.* The previous meta-analyses have reported the association of *VDR* gene polymorphisms with PCOS risk (14, 22). However, these analyses must be updated and performed on specific populations. It has been shown that the *VDR* polymorphisms may correspond to PCOS susceptibility in the Eastern Mediterranean Regional Office (EMRO) population, even though results are as yet uncertain and conflicting owing to the small sample size and limited statistical power (23-33). Appropriate and accurate data from developing countries in the EMRO are lacking; the majority of published data from these areas are outdated, heterogeneous, and suffering from limited sample sizes (34).

Consequently, the present study aimed to conduct an updated systematic review and meta-analysis to more reliably examine the association of *VDR* gene polymorphisms with susceptibility to PCOS in the EMRO population.

## 2. Materials and Methods

### Identification of eligible studies

The Preferred Reporting Items for Systematic Reviews and Meta-Analyses (PRISMA) guidelines were applied to conduct the present meta-analysis (35). An online search was carried out in PubMed, Scopus, SCI, and Google Scholar to retrieve related reports up to the end of 2020. The search strategy was based on terms and keywords including: `polycystic ovary syndrome OR PCOS', `vitamin D receptor OR VDR', and `polymorphisms OR variants'. Manual searches in the references cited within retrieved articles were also performed to select the relevant publications. Other prospective studies not included in the database were manually sought in the references of the obtained papers.

The inclusion criteria for this meta-analysis required that the articles be written in English, and included case-control studies, research on the association of ApaI, BsmI, FokI, and TaqI polymorphisms in the *VDR* gene with PCOS, investigations on the accessibility of genotype distributions in cases and controls for calculating odds ratios (ORs) and their 95% confidence intervals (CI), as well as studies on PCOS diagnosis using Rotterdam and National Institute of Child Health and Human Development criteria. Case reports, publications without full text, reviews, editorial comments, repeat publications, those with unavailability of the genotypic data, reports on the association of other polymorphisms in the *VDR* gene with PCOS, and the current meta-analysis did not include research that used an animal model. In the case of multiple publications reported by the same team, the most recent or largest sample sizes were chosen.

### Data extraction

The eligible studies were selected according to the following criteria by 2 independent authors (A.Az and A.SH), and any disagreements were decided by a third reviewer. The first author of each study, publication year, country of origin, the sample size of cases and controls, and data of the frequency genotype of distribution were recorded.

### Ethical considerations

The study was performed under the approval of the Ethical Committee of Kurdistan University Medical Sciences, Sanandaj, Iran (Code: IR.MUK.REC.1398.216).

### Statistical analysis

Using crude ORs and their 95% CI, the statistical power of the relationship between *VDR* gene variants and PCOS risk was determined. Allelic, recessive, and dominant genetic models were used in all analyses. A Chi-square test based on Q statistics was used to evaluate the inter-study heterogeneity, wherein the fixed model was applied when the p-value was > 0.10. Otherwise, a random-effects model was adopted. To examine which experiments had a substantial effect on the stability of the findings, one-way sensitivity tests were conducted. To evaluate possible publishing bias, we used the Begg funnel plot and Egger's linear regression test. STATA version 12.0 software (STATA Corporation, College Station, Texas, USA) was used to calculate the OR and 95% CI, and two-sided p-values < 0.05 were regarded as statistically significant.

## 3. Results

### Characteristics of eligible studies

**Figure 2:**
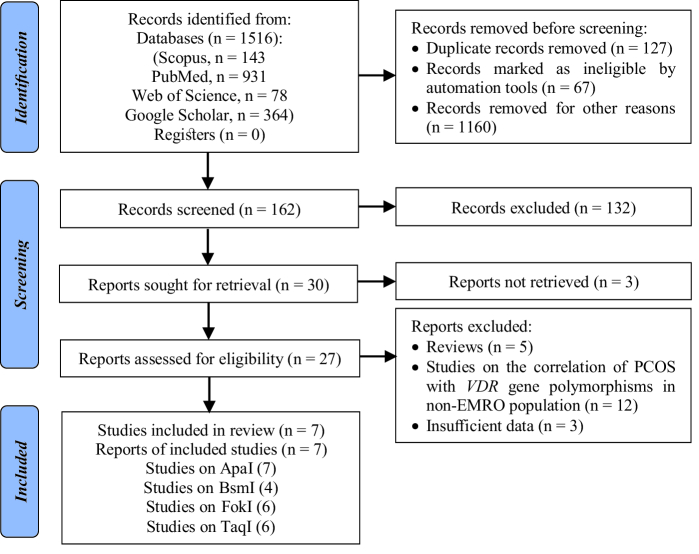
Flow chart of the literature retrieval and selection process.

The studies used in this meta-analysis were selected using the preferred reporting items for systematic reviews, and meta-analyses flow diagram, as seen in figure 2. To begin, we searched the abovementioned databases and found a total of 1,516 reports. The 1,354 duplicates and ineligible records were removed before screening, and reviews, irrelevant reports, and studies not considering the *VDR* gene or PCOS were excluded from the extracted records, which consisted of 152 papers. Then, we excluded 3 studies because there was inadequate evidence to quantify ORs and 95% CIs, or it was not a case-control design. Finally, this meta-analysis contained 7 articles conducted on the association of ApaI, BsmI, FokI, and TaqI polymorphisms in the *VDR* gene with risk of PCOS in the EMRO population.

The association of the *VDR* gene ApaI rs7975232 (G > T) polymorphism, BsmI rs1544410 (A > G) variant, the FokI rs2228570 (C > T) polymorphism, and Taq1 rs731236 (T > C) variant was examined in 6 (23, 24, 29, 30, 32, 33), 4 (26, 29-31), 6 (23, 24, 29-31, 33), and 4 (24, 28-30) case-control studies, respectively. Table I shows the characteristics of studies on *VDR* ApaI, BsmI, FokI, and TaqI variants and PCOS susceptibility.

**Table 1:** Characteristics of studies on *VDR* ApaI rs7975232 (A > C) variant and polycystic ovary syndrome susceptibility


		Genotype distribution ${}^{*}$					
**Author, yr (Ref)**	**Country**	**PCOS** **subjects**	**Healthy** **Controls**	<statement> <title>Case </title> </statement>			**Control**		
				**ApaI A/C (rs7975232)**				**AA**	**Aa**	**aa**	**AA**	**Aa**	**aa**
**El-shal ** ***et al.*** **, 2013 (32)**	Egypt	150	150	22 (14.6)	65 (43.4)	63 (42)	18 (12)	64 (42.6)	68 (45.4)
**Mahmoudi ** ***et al.*** **, 2015 (30) **	Iran	35	35	15 (42.9)	11 (31.4)	9 (25.7)	8 (22.9)	21 (60.0)	6 (17.1)
**Mahmoudi ** ***et al.*** **, 2009 (29) **	Iran	162	162	58 (35.8)	68 (42.0)	36 (22.2)	49 (30.2)	90 (55.6)	23 (14.2)
**Abdul-Hassan ** ***et al.*** **, 2017 (24) **	Iraq	50	50	1 (2)	28 (56)	21 (42)	6 (12)	23 (46)	21 (42)
**Humadi ** ***et al.*** **, 2018 (23) **	Iraq	100	100	60 (60)	24 (24)	16 (16)	40 (49)	2 (32)	28 (28)
**Al Thomal ** ***et al.*** **, 2018 (33)**	Saudi Arabia	17	16	1 (6.25)	7 (43.75)	8 (50)	2 (11.76)	8 (47.06)	7 (41.18)
**BsmI A/G (rs1544410)**				**BB**	**Bb**	**bb**	**BB**	**Bb**	**bb**
**Baqheri ** ***et al.*** **, 2013 (28)**	Iran	46	46	15 (32.6)	27 (58.7)	4 (8.7)	20 (43.5)	24 (52.2)	2 (4.35)
**Mahmoudi ** ***et al.*** **, 2015 (30)**	Iran	35	35	10 (28.6)	12 (34.3)	13 (37.1)	5 (14.3)	23 (65.7)	7 (20.0)
**Mahmoudi ** ***et al.*** **, 2009 (29)**	Iran	162	162	24 (14.8)	85 (52.5)	53 (32.7)	18 (11.1)	91 (56.2)	53 (32.7)
**Ramezani ** ***et al.*** **, 2020 (26)**	Iran	40	38	25 (65.8)	10 (26.3)	3 (7.9)	23 (57.5)	16 (40.0)	1 (2.5)
**Fok I C/T (rs2228570)**				**FF**	**Ff**	**ff**	**FF**	**Ff**	**ff**
**Baqheri ** ***et al.*** **, 2013 (28)**	Iran	46	46	4 (8.696)	20 (43.48)	22 (47.83)	2 (4.348)	15 (32.61)	29 (63.04)
**Mahmoudi ** ***et al.*** **, 2015 (30)**	Iran	35	35	16 (45.7)	17 (48.6)	2 (5.7)	24 (68.6)	10 (28.6)	1 (2.8)
**Mahmoudi ** ***et al.*** **, 2009 (29)**	Iran	162	162	83 (51.2)	67 (41.4)	12 (7.4)	96 (59.3)	59 (36.4)	7 (4.3)
**Abdul-Hassan ** ***et al.*** **, 2017 (24)**	Iraq	50	50	2 (4)	38 (76)	10 (20)	0 (0)	36 (72)	14 (28)
**Humadi ** ***et al.*** **, 2018 (23)**	Iraq	100	100	50 (50)	30 (30)	20 (20)	36 (36)	30 (30)	34 (34)
**Al Thomali ** ***et al.*** **, 2018 (33)**	Saudi Arabia	17	16	1 (6.25)	6 (37.50)	9 (56.25)	1 (5.88)	4 (23.53)	12 (70.59)
**TaqI T/C (rs731236)**				**TT**	**Tt**	**tt**	**TT**	**Tt**	**tt**
**Baqheri ** ***et al.*** **, 2013 (28)**	Iran	38	38	16 (42.11)	14 (36.84)	8 (21.05)	17 (44.74)	19 (50)	2 (5.26)
**Mahmoudi ** ***et al.*** **, 2015 (30)**	Iran	35	35	15 (42.9)	14 (40.0)	6 (17.1)	15 (42.9)	16 (45.7)	4 (11.1)
**Mahmoudi ** ***et al.*** **, 2009 (29)**	Iran	162	162	71 (43.8)	71 (43.8)	20 (12.4)	72 (44.4)	76 (46.9)	14 (8.6)
**Abdul-Hassan ** ***et al.*** **, 2017 (24)**	Iraq	50	50	1 (2)	39 (78)	10 (20)	6 (12)	37 (74)	7 (14)
**Al Thomali ** ***et al.*** **, 2018 (33)**	Saudi Arabia	17	16	4 (25)	8 (50)	4 (25)	6 (35.29)	7 (41.18)	4 (23.53)
**El-shal ** ***et al.*** **, 2013 (32)**	Egypt	150	150	40 (26.7)	74 (49.3)	36 (24)	69 (46)	61 (40.7)	20 (13.3)
*Data presented as frequency (Number [Percentage]). PCOS: Polycystic ovary syndrome, rs: Reference SNP cluster ID									

### Quantitative results 

Table II shows the details of the association of *VDR* gene ApaI rs7975232 (A > C), BsmI rs1544410 (A > G), FokI rs2228570 (C > T), and TaqI rs731236 (T > C) polymorphisms with PCOS predisposition. ApaI (C > A) correlates to ApaI (A/a), BsmI (A/G) to BsmI (B/b), FokI (C/T) to FokI (F/f), and TaqI (T > C) to TaqI (T/t), where the minor vs. major alleles were evaluated for their connection with the disease as a susceptible/protective factor.

**Table 2:** The detailed results of the association between *VDR* gene polymorphisms and risk of PCOS in the EMRO population


***VDR*** **-SNP**	**No. of studies**	**Q test P-value**	**I-squared%**	**Egger P-value**	**Model**	**OR (95% CI)**	**P-value**
**ApaI rs7975232 (A > C)**							
**A vs. a (allelic model)**	7	0.064	49.6	0.613	Fixed	1.120 (0.941-1.332)	0.20
**AA vs. aa (homozygote model)**	7	0.097	44.0	0.253	Fixed	1.167 (0.808-1.685)	0.41
**AA vs. Aa+aa (dominant model)**	7	0.146	37.1	0.201	Fixed	1.466 (1.093-1.967)	0.01
**aa vs. AA+Aa (recessive model)**	7	0.154	36.0	0.553	Fixed	1.033 (0.790-1.351)	0.81
**BsmI rs1544410 (A > G)**							
**B vs. b (allelic model)**	4	0.686	0.0	0.481	Fixed	0.994 (0.778-1.268)	0.95
**BB vs. bb (homozygote model)**	4	0.485	0.0	0.082	Fixed	1.039 (0.578-1.866)	0.89
**BB vs. Bb+bb (dominant model)**	4	0.281	21.6	0.717	Fixed	1.229 (0.806-1.874)	0.33
**bb vs. BB+Bb (recessive model)**	4	0.354	7.9	0.094	Fixed	1.223 (0.814-1.838)	0.33
**FokI rs2228570 (C > T)**							
**F vs. f (allelic model)**	6	0.006	69.4	0.895	Random	1.134 (0.738-1.742)	0.56
**FF vs. ff (homozygote model)**	6	0.106	45.0	0.876	Fixed	1.446 (0.865-0.2.417)	0.15
**FF vs. Ff+ff (dominant model)**	6	0.043	56.3	0.648	Random	1.012 (0.535-1.912)	0.97
**ff vs. FF+Ff (recessive model)**	6	0.296	18.1	0.328	Fixed	0.669 (0.455-0.982)	0.04
**TaqI rs731236 (T > C)**							
**T vs. t (allelic model)**	6	0.365	8.1	0.756	Fixed	0.715 (0.590-0.866)	< 0.001
**TT vs. tt (homozygote model)**	6	0.485	0.0	0.681	Fixed	0.435 (0.281-0.673)	< 0.001
**TT vs. Tt+tt (dominant model)**	6	0.107	44.7	0.689	Fixed	0.696 (0.524-0.923)	0.01
**tt vs. TT+Tt (recessive model)**	6	0.798	0.0	0.925	Fixed	1.791 (1.224-2.621)	< 0.001
*VDR*-SNP: Vitamin D receptor-single nucleotide polymorphism, rs: Reference SNP cluster ID, OR: Odds ratio, PCOS: Polycystic ovary syndrome, EMRO: Eastern Mediterranean Regional Office. I-squared-based Q statistics were used to evaluate the inter-study heterogeneity, wherein the fixed model was applied for conditions with no significant heterogeneity. Begg funnel plot and Egger's linear regression test were used to evaluate possible publishing bias. The pooled OR with 95% CI was calculated for the strength of the associations. Two-sided p-values < 0.05 were regarded as statistically significant							

#### *VDR* ApaI variant and PCOS risk

**Figure 3:**
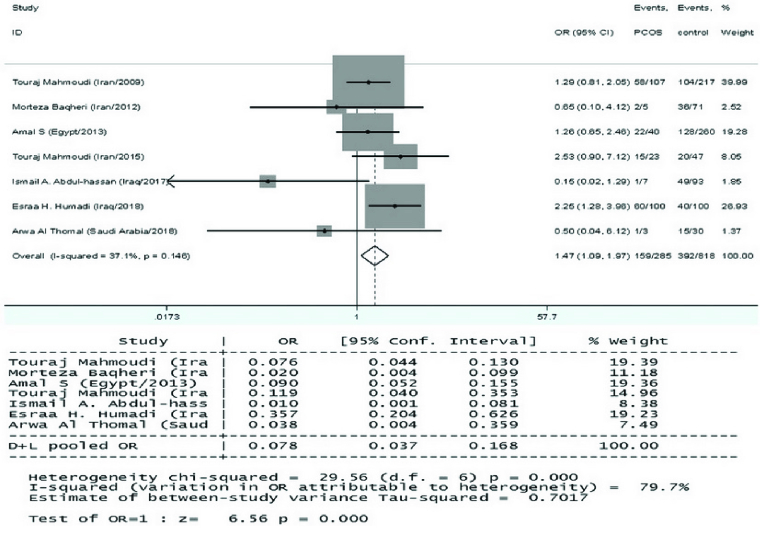
Forest plots of *VDR* ApaI (rs7975232) with dominant model: AA vs. Aa+aa and PCOS in the overall populations.

This review included 6 studies on the relationship between the *VDR* ApaI rs7975232 (A > C) polymorphism and risk of PCOS in the EMRO population. In all reports, the heterogeneity test showed no substantial heterogeneity. Therefore, the fixed effects model findings from the Mantel-Haenszel system were applied. As illustrated in figure 3, the correlation of ApaI SNP in the *VDR* gene with vulnerability to PCOS was statistically significant only in the dominant genetic model in the total populations (AA vs. Aa+aa: OR = 1.466, 95% CI = 1.093-1.9671, p = 0.01). No significant relationships of the *VDR* ApaI polymorphism with susceptibility to PCOS were observed in the other models (allelic, homozygote, and recessive genetic models, p > 0.05).

#### *VDR* BsmI variant and PCOS susceptibility

studies on the relationship between the *VDR* BsmI polymorphism and PCOS susceptibility were included in the meta-analysis (Table I). The heterogeneity test found that no substantial heterogeneity occurred in any of the included reports, and the fixed effects model results of the Mantel-Haenszel method were added to the analysis. There was no significant correlation between the *VDR* BsmI variant and PCOS susceptibility in the included EMRO population (p > 0.05).

#### *VDR* FokI variant and PCOS susceptibility

**Figure 4:**
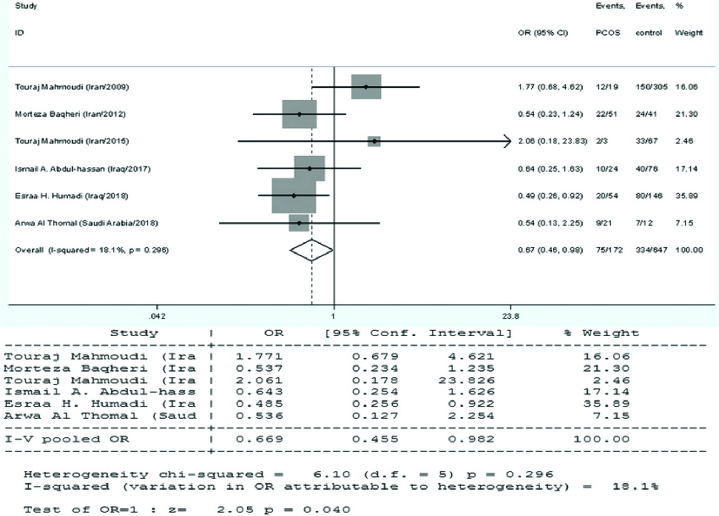
Forest plots of *VDR* FokI polymorphism (rs2228570) with recessive model: ff vs. FF+Ff and PCOS in the overall populations.

The current meta-analysis included a total of 8 reports on the relationship between the *VDR* FokI polymorphism and PCOS susceptibility, as seen in table I. We used the findings from the random-effects model on the grounds that heterogeneity was significant in certain model contrasts (allelic and homozygote models). A significant association of decreased risk was observed in the recessive genetic model (ff vs. FF+Ff: OR = 0.669, 95% CI = 0.455-0.982, p = 0.04) (Figure 4). In the allelic (F vs. f), homozygote (FF vs. ff), and dominant (FF vs. Ff+ff) genetic models, the pooled analysis found no significant association between this locus and PCOS risk (p > 0.05).

#### *VDR* TaqI variant and PCOS susceptibility

**Figure 5:**
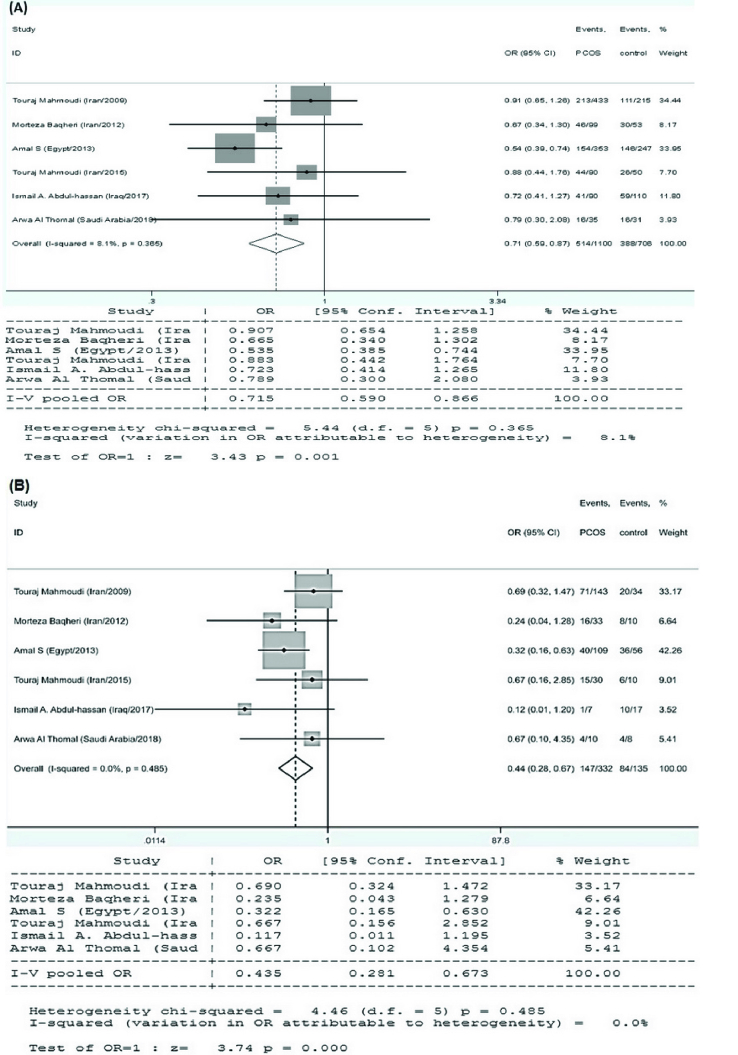
Statistical analysis of the association between *VDR* TaqI polymorphism and PCOS risk in the (A) T vs. t and (B) TT vs. tt model.

**Figure 6:**
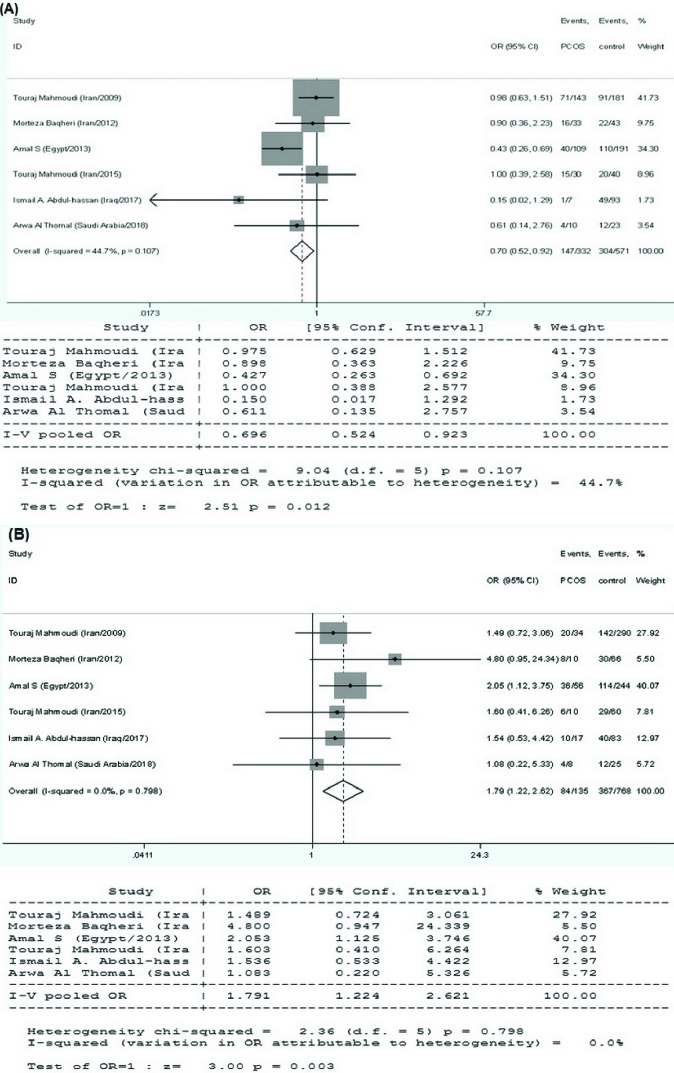
Statistical analysis of the association between *VDR* TaqI polymorphism and PCOS risk in the (A) TT vs. Tt + tt and (B) tt vs. TT + Tt model.

6 research papers on the association of the *VDR* TaqI polymorphisms with the risk of PCOS were found, as shown in table I. There was no significant heterogeneity in any of the comparisons, so the results of the random effects model were used in the analysis. In all 4 genetic models, there were significant associations of increased risk (T vs. t: OR = 0.715, 95% CI = 0.590-0.866, p < 0.001; TT vs. tt: OR = 0.435, 95% CI = 0.281-0.673, p < 0.001; TT vs. Tt+tt: OR = 0.696, 95% CI = 0.524-0.923, p = 0.01; tt vs. TT+Tt: OR = 1.791, 95% CI = 1.224-2.621, p < 0.001) in the included EMRO population (Table II, Figures 5 and 6).

### Publication bias

As indicated in table II, the Begg and Egger trials and the Begg funnel plots were carried out in all comparisons. No significant asymmetrical evidence was found, indicating that there was no publication bias in the meta-analysis.

## 4. Discussion 

In this meta-analysis, we presented the evidence on 4 *VDR* polymorphisms (ApaI, BsmI, FokI, and TaqI), and evaluated their association with PCOS risk in the EMRO population. The meta-analysis included a total of 7 articles based on 560 PCOS patients and 559 controls, which showed that the *VDR* TaqIpolymorphism in all comparisons consisting of allelic, homozygote, dominant, or recessive genetic models was associated with PCOS susceptibility. Furthermore, the *VDR* ApaI variant in the dominant model and *VDR* FokIpolymorphism in the recessive model were found to be significantly related to the risk of PCOS. However, the BsmI polymorphism was not significantly correlated with PCOS susceptibility in the included population.

Complex gene-gene and gene-environment interactions have been identified as major risk factors for the development of PCOS. PCOS is often associated with several complications, including hyperandrogenism, oligoanovulation, insulin tolerance, and metabolic anomalies. According to various reports, vitamin D deficiency has been linked to several metabolic risks in women with PCOS (36, 37). The active form of vitamin D exerts its effect by binding to *VDR*, as seen in figure 1. *VDR* gene variations have been linked to serum levels of insulin in women with PCOS in previous investigations (38). Serum 25-hydroxyvitamin D ([25 [[OH]] D) has been demonstrated to have a negative influence on VDR-mediated insulin resistance via regulating the expression of target genes (30). The *VDR* gene, which is involved in the insulin signaling system, could be a candidate risk factor for PCOS (39).

The polymorphisms ApaI, BsmI (intron 8), and TaqI (exon 9) are adjacent to the 3'-untranslated portion of the *VDR* gene, but they do not affect the *VDR* protein. ApaI, BsmI, and TaqI variants have also been shown to be in deep linkage disequilibrium (40). Even though these SNPs may not affect structural or protein expression, they can impact *VDR* transcript stability by adding changes in miRNA binding sites or other regulatory factors and gene expression while they are in linkage disequilibrium with different variants in the *VDR* gene's 3-untranslated region (41). Nevertheless, *VDR* mRNA produced by the t allele is more stable in peripheral blood mononuclear cells with the Tt TaqI haplotype than those with the T allele (42). Consequently, these *VDR* gene polymorphisms could be involved in the development of PCOS; nevertheless, since these polymorphisms are mostly nonfunctional, linkage imbalances with another undisclosed functional variant of the *VDR* gene seem to be the most plausible reason for the observed association.

In allele comparisons, the pooled results of the current meta-analysis revealed a substantial connection between the TaqI polymorphism and PCOS susceptibility. Our findings revealed a 0.715-, 0.435- and 0.696-fold lower risk for PCOS in the allelic, homozygote, and dominant models, respectively. Accordingly, the recessive genetic model of the TaqI variant (tt vs. TT+Tt) showed a 1.791-fold higher risk for PCOS. We compared minor alleles vs. major alleles in all analysis models. Our data indicated that the minor allele of the TaqI polymorphism (T allele) provides a protective genetic background against PCOS and the major allele (t allele) is a predisposing factor. Furthermore, the present meta-analysis showed that the ApaI variant is significantly associated with PCOS in the dominant genetic model by increasing the disease risk by 1.466-fold. This highlights the predisposing effect of the minor allele of the ApaI polymorphism. Lack of association for the other genetic models could be due to the quality and power of the included studies and their low sample sizes. Besides, we did not find any significant protective/predisposing association between the *VDR* BsmI variant and PCOS risk. The association of the TaqI variant with PCOS has been assessed in several studies.

Consistent with our results, a meta-analysis disclosed that the T allele of the *VDR* TaqISNP was significantly associated with PCOS risk. However, a correlation of *VDR* ApaI and BsmI with susceptibility to PCOS was not found in any of the included studies (43). In contrast to our findings, a meta-analysis published in 2019 found a link between ApaI and BsmI*,* and PCOS vulnerability in the Asian population. However, *VDR* TaqI did not show a connection (22). In a similar study, a group of researchers discovered an essential link between ApaI and PCOS vulnerability but no link between TaqI and risk of PCOS (14). However, they found only a small increase in PCOS risk associated with the BsmI A/G polymorphism in Asians based on ethnic diversity. Overall, this study's results are aligned with some studies (24, 26, 28, 32) and are in contrast to others (44-46), and might shed some light on how to proceed henceforth in determining the etiology of PCOS. The fact that the meta-analysis contained different studies, with different sample sizes and different statistical abilities may explain the contradictory findings.

Another of the *VDR* variants is FokI, which is located on exon 2 and results in a protein of different sizes. While this SNP has not been shown to interact with ligand binding, heterodimer formation with retinoid X receptor, or DNA binding, most studies have indicated that the shorter type of the protein (424 aa) is somewhat more active than the longer form (427 aa). However, it tends to be a gene- and cell-type-specific phenomenon, indicating that specific genes and cell types could be more susceptible to the polymorphism's effect than others (40, 47). According to some research, this polymorphism controls mRNA expression and may lead to different diseases (48). Although our study found that *VDR* FokI in the recessive genetic model (ff vs. FF+Ff) can influence PCOS, the other genetic models did not reveal significant protective/susceptible effects. Similar to our findings, 2 other meta-analyses also showed that *VDR* FokI (rs2228570) did not reveal a relationship with PCOS susceptibility (14, 22). The association of the FokI polymorphism with PCOS susceptibility has been examined in several case-control studies with varied results (23, 29, 31). These inconsistencies may be due to different sample sizes, included reports and levels of statistical power.

Despite the usefulness of the present meta-analysis (with its amalgamation of the key findings from different reports to provide a more comprehensive oversight of the issue at hand), we are aware of several limitations that should be addressed in interpreting our results. These are: the potential language bias from the exclusive focus on English-language publications; the fact that most of the studies were conducted in Iranian populations and the comparatively small number of studies examining the other EMRO populations; the lack of consideration for other factors potentially impacting PCOS susceptibility (such as age, gender, genetic variation, environmental factors); and the relatively small sample size.

## 5. Conclusion

In summary, the current meta-analysis provided statistical evidence that *VDR* ApaI in the dominant model, *VDR* FokI in the recessive model, and *VDR* TaqI polymorphisms in all genetic models are associated with PCOS susceptibility in the EMRO population. The BsmI variant did not reveal a relationship with PCOS susceptibility. However, these observations may not extend to other ethnic groups. Further analysis with a larger sample size and consideration of other confounding variables are needed before a firm conclusion can be drawn.

##  Conflict of Interest

The authors declare that there is no conflict of interest.
